# A novel methyl-binding domain protein enrichment method for identifying genome-wide tissue-specific DNA methylation from nanogram DNA samples

**DOI:** 10.1186/1756-8935-6-17

**Published:** 2013-06-07

**Authors:** Verity F Oliver, Jun Wan, Saurabh Agarwal, Donald J Zack, Jiang Qian, Shannath L Merbs

**Affiliations:** 1 Department of Ophthalmology, Johns Hopkins University School of Medicine, 600 North Wolfe Street, Baltimore, MD 21287, USA; 2 Ludwig Institute for Cancer Research, 9500 Gilman Drive, La Jolla, CA 92093-0653, USA; 3 Division of Biological Sciences, University of California, San Diego, 9500 Gilman Drive, La Jolla, CA 92093-0653, USA; 4 Department of Molecular Biology and Genetics, Johns Hopkins University School of Medicine, 600 North Wolfe Street, Baltimore, MD 21287, USA; 5 Department of Neuroscience, Johns Hopkins University School of Medicine, 600 North Wolfe Street, Baltimore, MD 21287, USA; 6 Institute of Genetic Medicine, Johns Hopkins University School of Medicine, 600 North Wolfe Street, Baltimore, MD 21287, USA; 7 Institut de la Vision, 17 rue Moreau, 75012, Paris, France

**Keywords:** DNA methylation, Tissue-specific differentially methylated regions, Retina, MBD, Nanogram

## Abstract

**Background:**

Growing evidence suggests that DNA methylation plays a role in tissue-specific differentiation. Current approaches to methylome analysis using enrichment with the methyl-binding domain protein (MBD) are restricted to large (≥1 μg) DNA samples, limiting the analysis of small tissue samples. Here we present a technique that enables characterization of genome-wide tissue-specific methylation patterns from nanogram quantities of DNA.

**Results:**

We have developed a methodology utilizing MBD2b/MBD3L1 enrichment for methylated DNA, kinase pre-treated ligation-mediated PCR amplification (MeKL) and hybridization to the comprehensive high-throughput array for relative methylation (CHARM) customized tiling arrays, which we termed MeKL-chip. Kinase modification in combination with the addition of PEG has increased ligation-mediated PCR amplification over 20-fold, enabling >400-fold amplification of starting DNA. We have shown that MeKL-chip can be applied to as little as 20 ng of DNA, enabling comprehensive analysis of small DNA samples. Applying MeKL-chip to the mouse retina (a limited tissue source) and brain, 2,498 tissue-specific differentially methylated regions (T-DMRs) were characterized. The top five T-DMRs (*Rgs20*, *Hes2*, *Nfic*, *Cckbr* and *Six3os1*) were validated by pyrosequencing.

**Conclusions:**

MeKL-chip enables genome-wide methylation analysis of nanogram quantities of DNA with a wide range of observed-to-expected CpG ratios due to the binding properties of the MBD2b/MBD3L1 protein complex. This methodology enabled the first analysis of genome-wide methylation in the mouse retina, characterizing novel T-DMRs.

## Background

DNA methylation is an epigenetic modification known to be important in many cellular processes, including tissue-specific gene expression. There is no standardized method for DNA methylation analysis and most array-based methods for genome-wide profiling require input DNA in microgram quantities, making them impractical for small tissue samples (Table 1). Sodium bisulfite modification is traditionally considered the gold-standard technique for assessing methylation [1]. Alternatives to bisulfite conversion include enrichment of methylated/unmethylated DNA by the use of methylation-sensitive restriction enzymes (MREs, for example, HpaII), immunoprecipitation with an anti-5-methylcytosine antibody (MeDIP) or affinity enrichment with methyl-binding domain proteins (MBDs) (Table 1). After bisulfite conversion or enrichment, DNA methylation can be quantified via next-generation sequencing or hybridization to a microarray. While whole genome bisulfite sequencing (WGBS) can quantify DNA methylation at single-nucleotide resolution using nanogram amounts of DNA, it requires robust bioinformatics resources, and the current cost-per-sample is still relatively high; thus, alternative methods to perform targeted bisulfite sequencing, such as reduced-representation bisulfite sequencing (RRBS) have been developed [2]. The single-nucleotide resolution of RRBS comes at the cost of reduced genomic coverage compared to MeDIP and MBD enrichment-based methods [3,4]. The most common MBD enrichment uses MBD2b, which shows high affinity for double-stranded, methylated DNA, in contrast to the MeDIP antibody, which binds methylated single-stranded DNA. The binding affinity of MBD2b is sequence independent, and MBD enrichment can be further enhanced by the addition of MBD3L1. Methylated-CpG island recovery assay (MIRA) [5] uses this protein complex and is now commercially available as a kit. Direct next-generation sequencing comparison has shown that MBD enrichment can identify more differentially methylated regions (DMRs) than either RRBS or MeDIP [3], but it is currently an underutilized methodology, particularly in combination with the more economical array-based analyses.

**Table 1:** Comparative DNA requirements for genome-wide methylation analysis platforms

**Methodology**	**Starting DNA required (μg)**	**Platform**
WGBS [6-8]	0.01–0.1	Illumina
MeDIP-Seq [3,4]	0.05–0.3	Illumina
MBD-Seq [3]	1^a^	Illumina
RRBS-Seq [3,9]	0.01–0.05	Illumina
MRE-Seq [10]	3	Illumina
MeDIP-Chip [11]	4	Affymetrix promoter array
MBD-Chip [11]	1	Affymetrix promoter array
MRE-Chip [12]	2	User defined
Infinium Methylation BeadChip [13]	0.8	Illumina^b^
CHARM [14]	10	NimbleGen
MeKL-Chip	0.02	NimbleGen/any

^a^Pooled four to eight 1-μg fractions.

^b^Not available for the mouse genome.

Methylation data obtained by hybridization to promoter, CpG island or CpG site-specific microarrays is biased and restricted by the design of the array platform. To overcome this bias, the comprehensive high-throughput array for relative methylation (CHARM) platform was developed to interrogate CpG sites genome-wide, irrespective of proximity to genes or CpG islands [14]. As the CHARM array was designed such that the genome coverage is driven by sequence and is not based on assumptions about CpG site location, it provides greater genome-wide coverage than other array platforms typically used for methylation analysis. The CHARM method enriches for unmethylated DNA using McrBC restriction enzyme digestion, which is compared to input DNA on a custom NimbleGen 2.1 million feature tiling array platform. Using this approach, tissue-specific differentially methylated regions (T-DMRs) have been detected in humans, mice and rats, including regions of low CpG density [15,16], which would normally be excluded from promoter and CpG island arrays. However, applicability of the McrBC-based CHARM protocol is limited because the enrichment protocol requires large amounts (10 μg) of starting genomic DNA (Table 1).

While MIRA can enrich DNA samples in the nanogram range [5], hybridization to the 2.1M NimbleGen microarray requires 1 μg of each DNA sample post-enrichment, necessitating high-magnitude whole genome amplification (WGA). There are two categories of commonly used WGA methodology: PCR amplification and isothermal DNA amplification. Ligation-mediated PCR (LM-PCR) [17] falls into the former category and involves the ligation of a unidirectional, double-stranded oligonucleotide universal adapter to blunted DNA fragments. The universal primer sequence can then be used to amplify all ligated fragments via PCR. LM-PCR enables amplification of a range of small PCR fragments (up to 2,000 bp in length), irrespective of the genomic sequence.

Currently, no protocols are available for MBD-chip with low amounts of starting DNA. With the goal of establishing a user-friendly method for assessing genome-wide DNA methylation in small (nanogram) DNA samples, we developed a new protocol based upon MBD2b/MBD3L1 enrichment [5] followed by amplification using modified LM-PCR, which we call MeKL-chip. The development of an array-based method for small tissue samples is advantageous for global methylation analysis because it does not have the computational burden of sequencing-based methods. Here we report on our MeKL-chip assay, its application to the mouse retina (a limited tissue source), and highlight its ability to detect novel T-DMRs with a wide range of CpG densities.

## Results

### Improved amplification efficiency by modified ligation-mediated PCR

**Figure 1:**
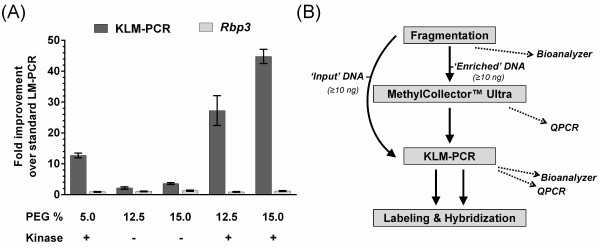
**Improvements achieved by the KLM-PCR protocol and an overview of the MeKL-chip method. (A)** Quantitative PCR assessment of fold increase in amplification using KLM-PCR with oligo_1 (dark grey) and *Rbp3* primers (light grey) as a direct comparison to the standard LM-PCR protocol (no kinase, 5% PEG-8000) using different amounts of PEG-8000 (%), with (+) and without (−) kinase treatment. The relative improvement of each reaction was calculated as a fold-change in comparison to the standard LM-PCR protocol. Amplification at *Rbp3* was used as a between-conditions loading control. Error bars based on standard error of the mean (*n* = 5). **(B)** Overview of the MeKL-chip method with the procedures for the input and enriched DNA as marked. The optional quality control steps are indicated by dashed arrows. KLM-PCR: kinase-modified ligation-mediated PCR; LM-PCR: ligation-mediated PCR; MeKL: MBD2b/MBD3L1 enrichment of DNA followed by KLM-PCR.

Using the published LM-PCR protocol as a starting point [17], we explicitly tailored our amplification for nanogram levels of input DNA. As LM-PCR can be limited by the efficiency of the ligation reaction, we made two modifications to improve the ligation reaction. Firstly, we increased the efficiency by adding a pre-ligation kinase treatment (kinase-modified ligation-mediated PCR, KLM-PCR) to repair DNA damage induced during fragmentation. Sonication can result in DNA breaks with 5′ hydroxyl and 3′ phosphate; treatment with T4 polynucleotide kinase reinstates the 5′ phosphate and 3′ hydroxyl moieties. This increased the amount of the template for ligation, improving the amplification 14-fold over the published method (Figure 1A). Additionally, we incorporated higher polyethylene glycol (PEG-8000) concentrations (12.5% or 15% vs 5%) in the ligation reaction buffer, as a previous report showed improved ligation efficiency under these conditions [18]. PEG-8000 concentrations of 12.5% and 15% increased the amplification two- to threefold, respectively (Figure 1A). Combined, these modifications increased the amplification 24- to 38-fold. Due to the difficulty of working with the viscous 15% PEG solution, we elected to use 13% PEG for subsequent experiments. To maximize PCR efficiency, a palindrome within the universal adapter sequence was disrupted to prevent the formation of an adapter-only template (Additional file 1: Figure S1A). We termed this combination of MBD2b/MBD3L1 enrichment [5] of DNA followed by KLM-PCR and subsequent hybridization to a microarray platform MeKL-chip (Figure 1B). MeKL can be used successfully to amplify sufficient DNA for microarray labeling and hybridization from as little as 10 ng of pre-enrichment, fragmented DNA (Additional file 1: Figure S1B).

### Identification of T-DMRs using MeKL-chip

**Figure 2:**
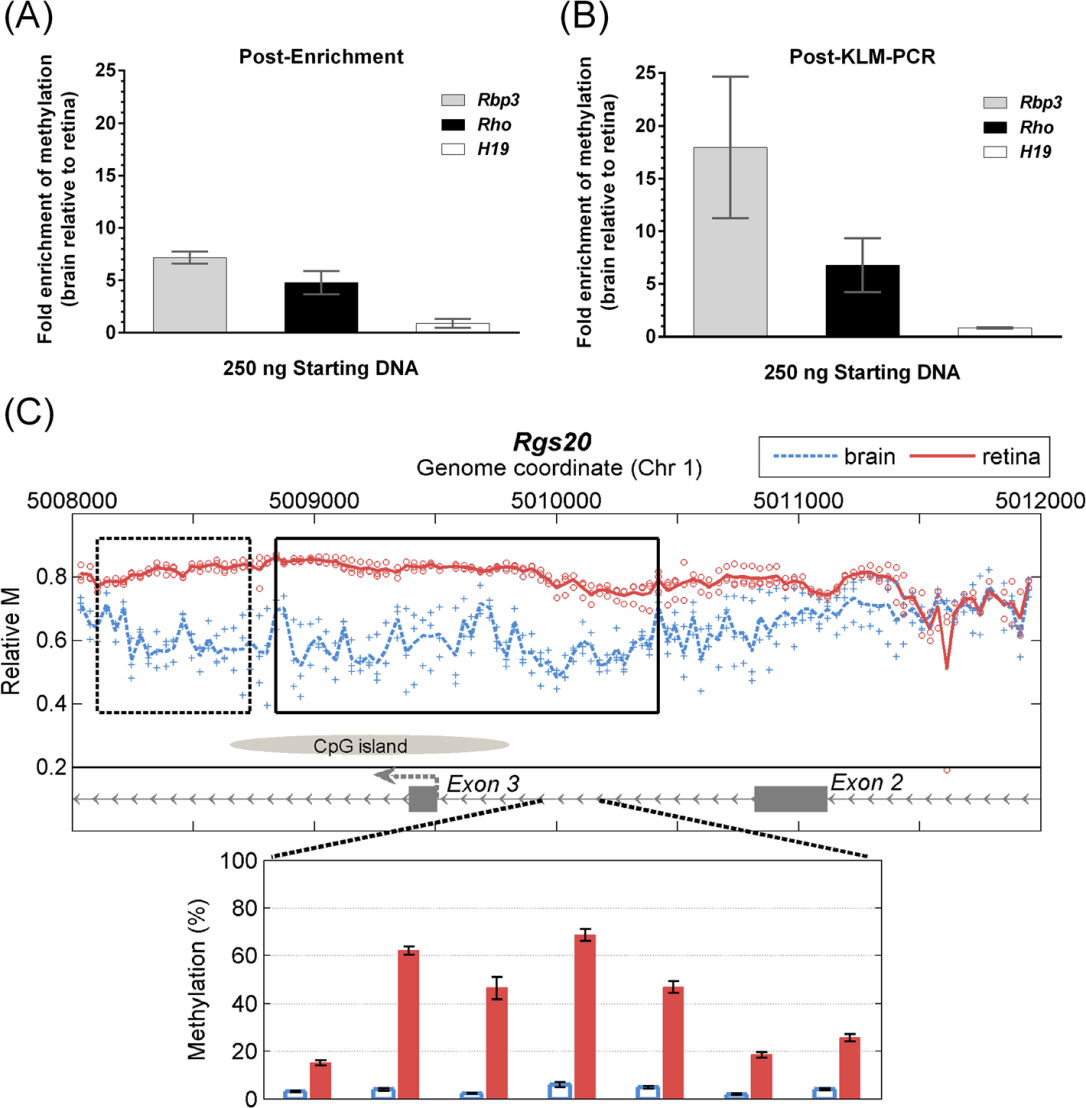
**Application of MeKL-chip to identify T-DMRs in the mouse retina and brain.** The fold-change between the retina and brain DNA samples was calculated and combined for samples used for MeKL-chip. The mean fold enrichment and standard error of the mean are plotted for triplicate enrichment experiments (three mice, three DNA samples). **(A)** Post-MBD enrichment, QPCR showed *Rbp3* (grey) and *Rho* (black) were enriched for methylated DNA in the brain samples as expected. Also as expected, no differential enrichment was evident for *H19* (white). **(B)** Post-KLM-PCR, QPCR showed maintenance of the enrichment pattern for methylation in the brain samples for *Rho* and *Rbp3*, while the amount of the *H19* template remained equal in both tissues. **(C)** Relative CpG methylation profiles as evaluated using MeKL-chip (top) and pyrosequencing validation (bottom) of the top T-DMR at *Rgs20* for the brain (blue, dashed) and retina (red, solid) from 250 ng of starting DNA. Each point is the relative methylation at one probe from one sample; blue and red lines show the average methylation of triplicates. Near the highest ranked T-DMR (black box, *P* < 10^-16^) is a second T-DMR (*P* < 0.0091, dashed box). The alternative TSS of the brain-specific isoform of *Rgs20* is indicated by a dashed arrow at Exon 3. Bisulfite pyrosequencing of seven CpGs within Intron 2 confirmed differential methylation between the mean of retina (red bars) and brain (blue bars) in five mice (Student’s two-tailed, paired t-test, *P* < 0.001). Error bars show the 95% CI (*n* = 5). KLM-PCR: kinase-modified ligation-mediated PCR; MBD: methyl-binding domain protein; MeKL: MBD2b/MBD3L1 enrichment of DNA followed by KLM-PCR; QPCR: quantitative PCR; T-DMR: tissue-specific differentially methylated region; TSS: transcription start site.

MeKL-chip was used to perform genome-wide profiling of adult mouse retinas to identify retinal T-DMRs, using the brain as a comparison neuronal tissue. As a quality control to demonstrate successful MBD2b/MBD3L1 enrichment of methylated DNA, quantitative PCR (QPCR) was performed at regions known to be differentially methylated between the retina and brain. The *retinol-binding protein 3* (*Rbp3*) and *rhodopsin (Rho*) genes are specifically expressed in retinal photoreceptors and have been shown to be hypomethylated in a cell-specific manner in the retina compared to non-retinal tissues [19]. In addition to QPCR validation of *Rho* and *Rbp3*, the imprinted gene *H19* (which is fully methylated on the paternal allele [20]) was also examined to ensure equal enrichment of methylated DNA in the retina and brain samples. After MBD enrichment, samples showed differential amplification of *Rho* and *Rbp3* in the brain compared to the retina, and a lack of differential enrichment of *H19* between the retina and brain (Figure 2A). The differential enrichment pattern between the retina and brain was maintained after KLM-PCR (Figure 2B). The amplified methylation-enriched and unenriched DNA from each sample were labeled and co-hybridized to the mouse CHARM array. In total, 2,498 novel T-DMRs were identified of which 1,449 were hypermethylated in the brain (Additional file 2: Table S1).

### Validation of potential T-DMRs by pyrosequencing

The top five T-DMRs were validated by pyrosequencing (Figure 2C and Additional file 3: Figure S2). The highest ranking T-DMR, which was hypermethylated in the retina, covered Exon 3 of *Rgs20* and its flanking introns (Figure 2C). An alternative transcription start site is located at Exon 3, an exon which is included in the brain-specific isoform of *Rgs20* and excluded from the retina-specific isoform [21]. The remaining top T-DMRs (associated with genes *Hes2*, *Nfic*, *Cckbr* and *Six3os1*) overlapped transcription start sites or were located intragenically (Additional file 3: Figure S2). The directionality of the differential methylation at all T-DMRs identified by MeKL-chip was completely consistent with the methylation levels observed in an independent mouse cohort by bisulfite pyrosequencing.

### Local CpG density and observed-to-expected CpG ratio analysis at T-DMRs

**Figure 3:**
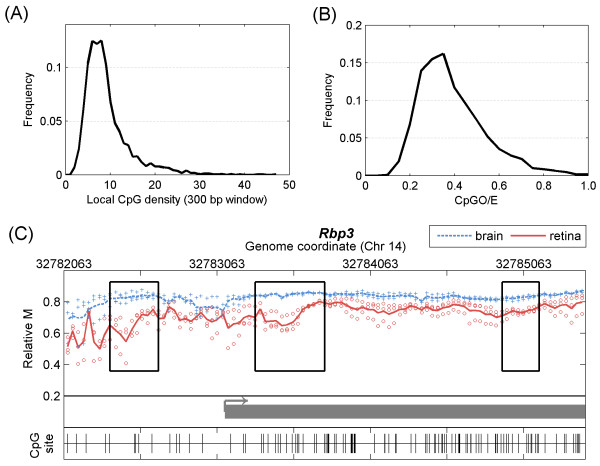
**MBD2 enrichment by MeKL-chip enables detection of differential methylation over a range of CpG densities and CpGO/Es. (A)** CpG density and **(B)** CpGO/E at each T-DMR was calculated over a 300 bp window. A range of CpG densities was observed, but the majority of T-DMRs were within low-CpG density regions. **(C)** Three T-DMRs (black boxes) associated with the retina-specific gene *Rbp3* for the retina (red, solid) and brain (blue, dashed) are shown. The TSS (arrow) and a portion of Exon 1 are shown in grey. The three T-DMRs had varying CpGO/Es: 0.31 (left box), 0.38 (central box) and 0.47 (right box), and local CpG densities: 4.6 (left box), 9.4 (center box) and 13.2 (right box). CpGO/E: observed-to-expected CpG ratio; MeKL: MBD2b/MBD3L1 enrichment of DNA followed by KLM-PCR; T-DMR: tissue-specific differentially methylated region; TSS: transcription start site.

To assess the properties of the MBD2b/MBD3L1 enrichment within the MeKL-chip assay, the CpG density and the observed-to-expected CpG ratios (CpGO/E) were determined for all T-DMRs identified. T-DMRs with a wide range of local CpG densities (1.7 to 46.2 in a 300 bp window) and CpGO/E (0.10 to 1.05) were isolated (Figure 3A,B). The majority of the T-DMRs were located in regions with relatively low CpG content (mean local CpG density of 9.2, mean CpGO/E of 0.39). An example of the diversity of CpG densities detectable within a genomic locus was found at the *Rbp3* gene, which is hypermethylated in brain tissue [19]. Three T-DMRs were identified within a 3,000 bp region of *Rbp3* with local CpG densities of 4.6, 9.4 and 13.2 and CpGO/Es of 0.31, 0.38 and 0.47, respectively (Figure 3C).

### MeKL-chip for nanogram DNA samples

**Figure 4:**
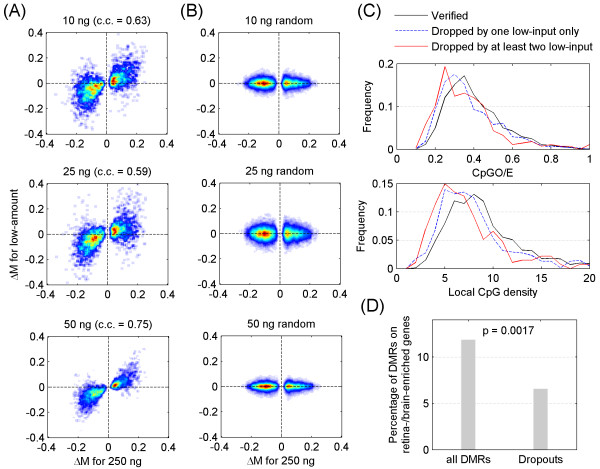
**Comparison of T-DMRs from high- and low-input samples identified using MeKL-chip. (A)** Correlation of differential methylation between retina and brain (ΔM) from high- and low-input arrays at T-DMRs initially identified within the 250-ng samples. **(B)** Relationship between ΔM of T-DMRs for high-input and that of randomly shuffled probes for the 10, 25 and 50 ng low-input arrays. **(C)** Distribution of CpGO/E (top panel) and CpG density (bottom panel) of verified T-DMRs for all three low-input arrays, dropouts from only one low-input array, and dropouts from at least two low-input arrays. **(D)** Percentage of T-DMRs and dropouts from at least two low-input arrays that were associated with retina-/brain-enriched genes identified from previously published exon array data. c.c: correlation coefficient; CpGO/E: observed-to-expected CpG ratio; MeKL: MBD2b/MBD3L1 enrichment of DNA followed by KLM-PCR; T-DMR: tissue-specific differentially methylated region.

To determine the effectiveness of MeKL-chip on low-input DNA samples, differing amounts of total starting DNA were fragmented, enriched and amplified from mouse retina and brain. Successful fragmentation was achieved with 50, 125 and 250 ng of sample DNA (data not shown). MBD2b/MBD3L1 enrichment was achieved using as little as 10 ng of fragmented sample DNA (Additional file 4: Figure S3A). Enrichment was maintained after KLM-PCR amplification in all low-input amounts examined (Additional file 4: Figure S3B). MeKL-chip data of the *Rgs20* region from the 10-ng input samples from the retina and brain revealed tissue-specific methylation differences comparable to those detected in the 250-ng arrays (Additional file 5: Figure S4). Array hybridization of the 10, 25 and 50 ng fragmented and MBD2b/MBD3L1-enriched DNA from retina and brain samples yielded analyzable results that were combined to provide a low-input MeKL-chip group. The difference in methylation between retina and brain (ΔM) at T-DMRs identified within the high-input samples (250 ng) was compared to the corresponding ΔM of these same T-DMRs from the low-input samples (10, 25 and 50 ng; Figure 4A). A strong correlation was observed between the high- and low-input results (correlation coefficient [c.c.] = 0.63, 0.59 and 0.75, respectively). In comparison, there was no correlation between high- and low-input experiments when the probe positions were randomly shuffled (Figure 4B). Over 61% of the 2,498 T-DMRs identified in the high-input samples had the same directionality of ΔM for all three low-input samples, which were termed ‘verified’ T-DMRs. The remaining T-DMRs showed inconsistent ΔM directions between the high- and low-input groups (visible in the upper left and lower right quadrants of Figure 4A), and were referred to as ‘dropouts.’ Notably, the majority (72%) of these dropout T-DMRs had an opposite ΔM direction in only one of the three low-input samples and less than 11% of all 2,498 T-DMRs had a conflicting ΔM direction with two or more low-input samples. The dropouts tended to have lower CpGO/E and lower CpG density (Figure 4C). Integrative analysis of the T-DMR data with exon array data in the same mouse tissues [22] revealed that only 6% of these dropouts were associated with retina-/brain-enriched genes when filtering for those dropouts absent from at least two low-input arrays (Figure 4D). These results indicated that the low-input arrays were able to detect differential methylation within biologically relevant regions and validated the use of the MeKL-chip method on low-input (≤50 ng) DNA samples.

## Discussion

We have demonstrated a novel combination of MBD2b/MBD3L1 enrichment, modified ligation-mediated PCR amplification and microarray hybridization, in a method termed MeKL-chip. Using our unique protocol, we have shown that MeKL-chip permits genome-wide DNA methylation analysis with as little as 20 ng of DNA (comprising 10 ng for enrichment and 10 ng input for comparison). The ability to study genome-wide methylation in small samples enabled us to identify novel T-DMRs in a limited tissue sample: the mouse retina. The MeKL-chip protocol fills a void in the currently available methylation profiling technologies, enabling regional examination of methylation patterns with a low computational requirement.

Ligation-mediated PCR (LM-PCR) is an approach well suited to the amplification of small DNA fragments [17]. In LM-PCR, universal linkers are ligated to blunt-ended DNA fragments, which are then used to amplify the ligated DNA via PCR [17]. We modified the LM-PCR protocol to include a pre-ligation kinase step to repair the fragmented DNA, included PEG during the ligation to increase ligation efficiency and modified the linker sequence to remove a palindrome. After these modifications, KLM-PCR was able to produce sufficient DNA for microarray hybridization (a minimum of 70-fold amplification) from post-enrichment samples derived from as little as 10 ng.

The top five T-DMRs identified by MeKL-chip and validated by pyrosequencing were all genes with known biological relevance in the retina. The top T-DMR at *Rgs20* (*regulator of G protein signaling 20*, Gene ID: 58175) overlapped an alternative transcription start site [21], which suggests that DNA methylation may play an essential role in the tissue-specific expression of *Rgs20* transcripts in the retina and brain. The location of the T-DMR within *Rgs20* is consistent with existing results that have shown a role for intragenic DNA methylation in tissue-specific expression mediated by alternative transcriptional start sites [23]. The transcription factor *Hes2* (*hairy and enhancer of split 2*, 15206) has been previously implicated in cell fate determination of the *Xenopus* retina [24], and *Six3os1*(100043902), a long non-coding RNA on the opposite strand to the *sine oculis-related homeobox 3* transcription factor, has also been described as important during retinal development [25]. *Cckbr* (*cholecystokinin B receptor*, 12426) mRNA has been reported in the rat retina [26]. *Nfic* (*nuclear factor I/C*, 18029) has been described as overexpressed in the retina of patients with proliferative vitreoretinopathy, a disease where aberrant wound healing occurs in the retina [27]. The ability to analyze global DNA methylation in the mouse retina has identified T-DMRs at biologically relevant genes in addition to novel targets that warrant further study. The vast majority of these top T-DMRs identified in the high-input (250 ng) arrays were also identified in the low-input (≤50 ng) arrays.

We observed that the overall difference in methylation at the T-DMRs was greater by pyrosequencing than by MeKL-chip. This difference in percentage methylation between array hybridization and pyrosequencing has been noted previously [15], suggesting that hybridization to CHARM arrays using either unmethylated-enriched or methylated-enriched DNA may underestimate the relative difference in methylation between samples as a consequence of the fixed dynamic range of microarrays.

One of the main advantages of using MBD2b/MBD3L1 enrichment, due to the unique binding properties of the MBD2b/MBD3L1 complex, is the ability to detect methylation within a wide range of CpG densities [11]. Although MeKL-chip is unable to measure site-specific CpG methylation compared to bisulfite-based sequencing methods, we successfully identified robust T-DMRs throughout the mouse genome. We were able to isolate T-DMRs with a broad range of observed-to-expected CpG ratios and CpG densities unlike the more frequently used MeDIP technique, which is known to be most efficient at low CpG density ranges [11]. Although the CpGO/Es of our T-DMRs ranged from 0.10 to 1.05, the majority of our T-DMRs had CpGO/Es less than that of a CpG island (CpGO/E > 0.6). This observation supports previously published data indicating that the majority of T-DMRs are identified in regions outside of CpG islands with relatively low CpG density, for example, CpG shores and CpG shelves [15,16,28,29]. While we selected the NimbleGen CHARM 2.1M platform because regions of low CpG density were incorporated into the array design, the MeKL-chip methodology is amendable to any array platform. The incorporation of PEG during ligation should facilitate next-generation sequencing library preparation from nanogram amounts of methylation-enriched DNA.

## Conclusions

We have demonstrated that the KLM-PCR method of WGA results in greater than 400-fold amplification of low-input DNA samples. This protocol has many potential downstream applications, including hybridization to custom arrays as well as next-generation sequencing-based platforms. By combining KLM-PCR WGA and MBD-affinity methylation enrichment with hybridization to a custom CHARM microarray (MeKL-chip), we were able to achieve robust identification of T-DMRs between the retina and brain within biologically relevant genes using nanogram quantities of input DNA. The MeKL-chip method enables genome-wide assessment of methylation in samples previously considered below the threshold for array-based, global methylation analyses. This methodology will be particularly useful for the identification of regional methylation differences between small tissue samples, for example, laser-capture microdissection collected DNA, and in detecting disease-associated methylation differences within affected cell layers.

## Methods

### Animals

Eight-week-old C57BL/6J male mice (*n* = 5) (Jackson Laboratories) were euthanized using IsoSol™ (VEDCO) exposure followed by neck dislocation. A second cohort of eight-week-old C57/B6J male mice (*n* = 5) was processed in an identical manner for validation using bisulfite pyrosequencing. All procedures were approved by the Johns Hopkins University Institutional Animal Care and Use Committee (IACUC) and were performed in accordance with guidelines in the National Research Council’s *Guide for the Care and Use of Laboratory Animals*.

### Samples

After euthanasia, the mouse eyes were immediately enucleated and placed into 1X PBS buffer. The cornea and lens were discarded and the eyecup placed into 500 μL of 1X Hanks’ balanced salt solution (HBSS, Invitrogen). The eyecup was incubated for 15 min at 37°C and then microdissected in 1X HBSS. Surrounding sclera was removed from the retina, and any remnants of retinal pigment epithelium were removed by gentle scraping. Three samples of 25 mg of brain cortex were removed from each mouse. DNA extraction was performed on each sample using the DNeasy Blood and Tissue kit (Qiagen) following the manufacturer’s protocols with addition of RNase A. DNA was eluted in 400 μL Buffer AE. Ethanol precipitation of the DNA samples was performed [30] and the DNA resuspended in 40 μL 1X TE pH 8.0. Retina/brain DNA samples originating from the same mouse were pooled. The amount of DNA in each sample was quantified using the Quant-iT™ PicoGreen® dsDNA Assay Kit (Invitrogen).

### MBD enrichment for methylated DNA

Prior to enrichment, 2.5 μg of DNA in 100 μL 1X TE pH 8.0 was fragmented to an average target size of 300 bp (duty cycle 10%, intensity 4, cycles per burst 200, time 60 sec) using a Covaris™ S220 Ultrasonicator. For smaller amounts of DNA, 50, 125 or 250 ng of DNA was fragmented in 50 μL (duty cycle 10%, intensity 5, cycles per burst 200, time 50 sec). The accuracy of the fragmentation was checked using an Agilent 2100 Bioanalyzer with the DNA 1000 kit. Enrichment was performed using the MethylCollector Ultra Kit (Active Motif) according to the manufacturer’s protocols (version C1) using Low-Salt Binding Buffer AM12. DNA cleanup after enrichment was performed using the MinElute PCR Purification kit (Qiagen). The enriched DNA was eluted in 10 μL Buffer EB. To enable quality control experiments, fragmented DNA was enriched in duplicate and the duplicate samples combined.

### QPCR validation of enrichment

QPCR was performed for the known brain/retina differentially methylated genes *Rho* (Gene ID 212541) and *Rbp3* (19661) [19] and a known equally methylated gene, *H19* (14955) [20]. QPCR was performed on an iQ™5 instrument (Bio-Rad) using 1X EvaGreen® dye (Biotium), Fermentas Maxima™ Hot Start Taq DNA Polymerase (Thermo Fisher Scientific) and 1.2 μL of 5 μM forward and reverse primer mix in a final 20 μL reaction volume. Next, 10 ng of fragmented, unenriched DNA, 1 μL of post-enrichment DNA or 10 ng post-amplification DNA were amplified in triplicate for the same sample at all three genes. The primer sequences were (5′ to 3′): *Rho* forward: AAGCAGCCTTGGTCTCTGTC, *Rho* reverse: CCCTCTGTGCCGTTCATGG, *Rbp3* forward: GGCCCAGATACAGAGGAACA, *Rbp3* reverse: GCTCGCTCAGTACCTCTTGG, *H19* forward: TGTGTAAAGACCAGGGTTGC and *H19* reverse: GGGAGAAAACTCAATCAGTTGC. The QPCR cycling conditions were: 95°C for 3 min, 50 × (95°C for 10 sec, 66.4°C for 30 sec, 72°C for 30 sec) followed by the standard dissociation steps. The mean threshold cycle (Ct) for each sample was used to calculate ΔCt_(retina-brain)_, and ΔCt was then used to calculate the fold enrichment for methylation (2^ΔCt^). A lower Ct for the brain sample and a fold enrichment greater than 2 should be observed post-enrichment as the brain is hypermethylated at *Rho* and *Rbp3*[19].

### Whole genome amplification by kinase ligation-mediated PCR

All buffers and enzymes used for amplification were from New England Biolabs (NEB) unless otherwise stated. Based on the LM-PCR protocol developed by the Ren laboratory [17], 5 μg of sonicated DNA was treated with RNAse A followed by phenol:chloroform:isoamyl alcohol extraction and purification with the MinElute PCR Purification kit. Next 500 ng of the sonicated DNA was treated with either 60 U of T4 Polynucleotide Kinase or mock treated (no kinase added) in a final volume of 300 μL of 1X T4 DNA ligase buffer and 100 μg/mL BSA for 2 h at 37°C. To fill in the fragmented ends, 200 μL of cold blunting mix containing 25 μL NEBuffer 3, 25 μL of dNTP mix (10 mM each dNTP), 2 μL 10 mg/ml BSA and 7.5 U of T4 DNA polymerase were added to each reaction and incubated at 12°C for 30 min. Reactions were extracted with phenol:chloroform:isoamyl alcohol, precipitated with 1 volume of isopropanol in the presence of 300 mM sodium acetate (pH 5.2) at −80°C overnight, washed twice with 70% ethanol and resuspended in 500 μL of H_2_O. Modified linkers (KLM-PCR Oligo_1: 5´ GCG GTG ACC CGG GAG ATC TGA GTT C 3´, Oligo_2: 5´ GAA CTC AGA TC 3´) [17] included a single base change (GAATTC to GAGTTC) in KLM-PCR Oligo_1, which disrupted the GAATTC palindrome thus improving the efficiency of the PCR. To anneal the linkers, 510 μL of 40 μM Oligo_1 and 490 μL of 40 μM Oligo_2 were combined with 250 μL of 5X Duplex Buffer (100 mM Tris–HCl pH 8.0, 0.1 mM EDTA, 250 mM NaCl, 25 mM β-mercaptoethanol) and aliquoted into 100 μL in PCR tubes. To test the efficiency of ligation under different concentrations of PEG-8000 [18], ligations were set up using 20 ng DNA from T4 Polynucleotide Kinase treated/untreated samples with 5%, 12.5% and 15% PEG-8000 using T4 DNA Ligase Buffer, 50% PEG-8000 solution, BSA (15 μg), T4 DNA Ligase (400 U) and modified, annealed linkers (75 μM), and were incubated at 16°C for 16 h followed by purification using the MinElute PCR Purification kit. The following steps were performed in a thermocycler: 95°C for 3 min, 55°C for 1 min, 0.1°C/sec to 48°C, 48°C for 3 min followed by 2°C decrements in temperature, holding for 3 min at each step until 4°C was reached. Real-time PCR of the ligation reactions was performed using iQ™ SYBR® Green Supermix (Bio-Rad). Taq DNA Polymerase and PfuTurbo® DNA polymerase (Agilent) were added to the supermix to reproduce the LM-PCR conditions, as LM-PCR required a non-hot-start DNA polymerase for initial fill-in of ligated products. QPCR was performed in triplicate using KLM-PCR Oligo_1 and in duplicate using a gene specific (*Rbp3*) primer pair as a loading control for DNA in each reaction. The cycling conditions were 72°C for 3 min for initial fill-in, 94°C for 2 min for initial denaturation, followed by 35 cycles of (94°C for 15 sec, 95°C for 15 sec, 60°C for 30 sec, 72°C for 30 sec and 72°C for 30 sec for image capture) and a disassociation curve. The relative fold-change between the various ligation conditions (+kinase vs kinase at 5% PEG, 12.5% PEG vs 5% PEG, 15% PEG vs 5% PEG, 12.5% PEG/+kinase vs 5% PEG/-kinase and 15% PEG/+kinase vs 5% PEG/-kinase) were calculated and plotted for five replicate experiments, along with the standard error of the mean (Figure 1A). For the microarray samples, the KLM-PCR conditions included phosphorylation of DNA by T4 Polynucleotide Kinase prior to the blunting reaction (see above), modified linkers and 13% PEG-8000 instead of 5% PEG-8000. Either 10 ng of fragmented DNA (input) or 10 μL of methylation-enriched DNA in 100 μL Buffer EB (Qiagen) was treated with 10 U of T4 polynucleotide kinase in 150 μL of 1X T4 DNA ligase buffer. After 1 h incubation at 37°C, 1 U of T4 DNA polymerase, 10 μL of 10X NEBuffer 3, 10 μL 10 mM dNTPs and 2 μL 10 mg/ml BSA in a volume of 50 μL were added to a total volume of 200 μL and reactions were incubated at 12°C for 20 min. Amplified DNA was quantified using a NanoDrop (Additional file 1: Figure S1).

### Microarray labeling and hybridization

Fragmented (input) DNA was labeled with Cy3, and enriched (methylated) DNA was labeled with Cy5, using the NimbleGen Dual-Color DNA Labeling kit (Roche) according to the manufacturers’ instructions. Samples were hybridized to the custom NimbleGen 2.1M feature mouse CHARM microarray at the Johns Hopkins Medical Institutions Deep Sequencing & Microarray Core Facility or the Johns Hopkins Bloomberg School of Public Health Genomic Analysis and Sequencing Core Facility.

### MeKL-chip data analysis

Analysis of the MeKL-chip data was performed using the R/Bioconductor software for CHARM as previously published [14,31]. In brief, this method used genome-weighted smoothing of probes within genomic regions to identify T-DMRs. Results from each NimbleGen CHARM array contained two sets of raw data: input (untreated) DNA and methyl-enriched DNA. Hybridization quality was assessed by a signal score, which examined the number of untreated channel signal probes that ranked above the background (anti-genomic control) probes. Successful hybridization was indicated by a higher signal score (usually greater than 0.85). After Loess normalization within samples for all control probes [31] and quantile normalization between samples had been performed, the relative methylation level for each probe was calculated as the ratio of the methylated probe to the input probe signal. As previously described [14], a t-test was adopted to identify differentially methylated probes between brain and retina samples (from triplicate arrays). Triplicate arrays consisted of either high-input (250 ng) or low-input (≤50 ng) retina and brain groups. The t-statistic cutoff in this study was set as *P* < 0.005. Consequently, T-DMRs were constituted by neighboring differentially methylated probes. T-DMRs with less than three probes were excluded from further analysis.

The definition of local CpG density by Pelizzola et al. [32] was adopted to analyze the identified T-DMRs. In general, the local CpG density of one nucleotide represents a weighted (0 at the boundary and 1 at the center) count of CpG sites surrounding the nucleotide, that is, 300 bp upstream and downstream from the nucleotide of interest (a window size selected based on the average fragment size). The local CpG density of a T-DMR was then obtained as mean value of local CpG densities of all nucleotides within the T-DMR. In addition, the observed-to-expected CpG ratio (CpGO/E) of a T-DMR was calculated using the standard formula:

$$\begin{array}{cc} \mathrm{CpGO}/E & =\frac{N_{\mathrm{CpG}}}{N_{C}\times N_{G}} \end{array}\times L,$$

where *N*_*CpG*_, *N*_*C*_ and *N*_*G*_ are the number of CpGs, and nucleotide Cs and Gs, respectively, and *L* is the length of the T-DMR sequence.

### Pyrosequencing

Using the EZ DNA Methylation-Gold™ Kit (Zymo), 1 μg of genomic DNA was bisulfite converted. Bisulfite-converted DNA was eluted twice in 10 μL M-Elution buffer and stored as 5 μL aliquots at −80°C. Genomic sequences surrounding the RefSeq genes were obtained using the UCSC Genome Browser for *Rgs20* (Gene ID: 58175), *Hes2* (15206), *Nfic* (18029), *Cckbr* (12426) and *Six3os1* (100043902). Pyrosequencing primers were designed (Additional file 2: Tables S2 and S3) within the identified DMR locations using the PyroMark Assay Design Software (Qiagen). PCR was performed using 1 μL of bisulfite-converted DNA and HotStarTaq DNA Polymerase (Qiagen) under the following cycling conditions: 95°C for 15 min; 45 cycles of (94°C for 30 s, annealing temperature from Additional file 2: Table S2 for 30 s, 72°C for 60 s); 72°C for 3 min; 4°C hold followed by storage at −20°C. Amplicons were analyzed on a PyroMark Q24 Pyrosequencer as per the manufacturer’s protocols and methylation at the CpG sites was quantified using the PyroMark Q24 software version 2.0.6.

## Abbreviations

c.c: Correlation coefficient; CHARM: Comprehensive high-throughput array for relative methylation; CpGO/E: Observed-to-expected CpG ratio; Ct: Threshold cycle; DMR: Differentially methylated region; dNTP: Deoxyribonucleotide triphosphates; HBSS: Hanks’ balanced salt solution; KLM-PCR: Kinase-modified ligation-mediated PCR; LM-PCR: Ligation-mediated PCR; MBD: Methyl-binding domain protein; MeDIP: Methylated DNA immunoprecipitation; MeKL: MBD2b/MBD3L1 enrichment of DNA followed by KLM-PCR; MIRA: Methylated-CpG island recovery assay; MRE: Methylation-sensitive restriction enzyme; PCR: Polymerase chain reaction; PEG: Polyethylene glycol; QPCR: Quantitative PCR; RRBS: Reduced-representation bisulfite sequencing; T-DMR: Tissue-specific differentially methylated region; TSS: Transcription start site; WGA: Whole genome amplification; WGBS: Whole genome bisulfite sequencing.

## Competing interests

The authors declare that they have no competing interests.

## Authors’ contributions

SLM, DJZ, VFO and SA designed the study. VFO performed the experiments. JW and JQ designed and performed the bioinformatics analysis. VFO, JW, SA, DJZ, JQ and SLM wrote the manuscript. DJZ, JQ and SLM supervised the project. All authors read and approved the final manuscript.

## Supplementary Material

Additional file 1: Figure S1 The KLM-PCR protocol. **(A)** Modification of the universal adapter oligo sequence. The original LM-PCR oligo contained a palindrome at the 3′ end [17]. Prevention of dimerization through the disruption of the oligo palindrome increases the amount of available oligo for KLM-PCR amplification. dNTPs are now the limiting factor in the amplification. **(B)** NanoDrop quantification of the mean micrograms of DNA produced after KLM-PCR. Bars represent the mean of duplicate experiments from amplification of 10, 25, 50 and 250 ng amounts of pre-enriched starting DNA, or 10 ng of unenriched (UE) DNA. Error bars show standard deviation (*n* = 2). Click here for file

Additional file 2: Table S1 Top 20 differentially methylated regions identified between mouse retina and brain from 250 ng of starting DNA. **Table S2.** PCR amplification primers used for pyrosequencing the top five DMRs identified using MeKL-chip. **Table S3.** Sequencing primers used for pyrosequencing validation of the top five DMRs identified using MeKL-chip. Click here for file

Additional file 3: Figure S2 Relative CpG methylation in the retina (red) and brain (blue) of the four other top T-DMRs (black boxes) evaluated using MeKL-chip (top plots) and pyrosequencing validation of the differential methylation (bottom graphs). See Figure 2C for description of MeKL-chip results. Pyrosequencing of CpGs within the T-DMR confirmed differential methylation (*P* < 0.001, Student’s two-tailed, paired t-test) between the retina (red bars) and brain (blue bars) in a second cohort of mice. Error bars show the 95% CI (*n* = 5). Click here for file

Additional file 4: Figure S3 Pre-hybridization validation of enrichment from low-input (10, 25 and 50 ng) enriched DNA from retina and brain samples. The mixed effects regression model with a random intercept for the measures from triplicate QPCR of two PCR amplifications of the same sample were used to calculate the mean difference and standard error in fold enrichment. **(A)** Post-enrichment, *Rbp3* (grey bars) and *Rho* (black bars) were enriched for methylated DNA in the brain samples for all amounts of starting DNA, whereas no enrichment was observed in unenriched (UE) DNA. **(B)** Post-KLM-PCR, QPCR showed maintenance of the enrichment pattern for methylation in the brain samples for *Rho* (black bars) and *Rbp3* (grey bars) and no enrichment of the UE samples. Click here for file

Additional file 5: Figure S4 MeKL-chip CpG site methylation profiles of the *Rgs20* region identified as a T-DMR (highest ranked, *P* < 10^-16^, black box; lower ranked, *P* < 0.0091, dashed box) for the 250-ng high-input samples in the brain (blue) and retina (red) (top plot) as previously shown in Figure 2. The 10 ng low-input sample at the same region of *Rgs20* is shown for direct comparison (lower plot) in brain (blue) and retina (red). Each point is the relative percentage methylation for 1 probe in 1 sample. The 250 ng plot contains biological triplicates and blue and red lines show the average methylation. The 10 ng plot contains one biological sample. The T-DMRs are still detectable in the 10 ng low-input sample. Click here for file
