# Supplementary figures and images for: A novel methyl-binding domain protein enrichment method for identifying genome-wide tissue-specific DNA methylation from nanogram DNA samples
Source: Epigenetics Chromatin. 2013 Jun 7;6:17. doi: 10.1186/1756-8935-6-17 (PMC3680319; doi:10.1186/1756-8935-6-17)

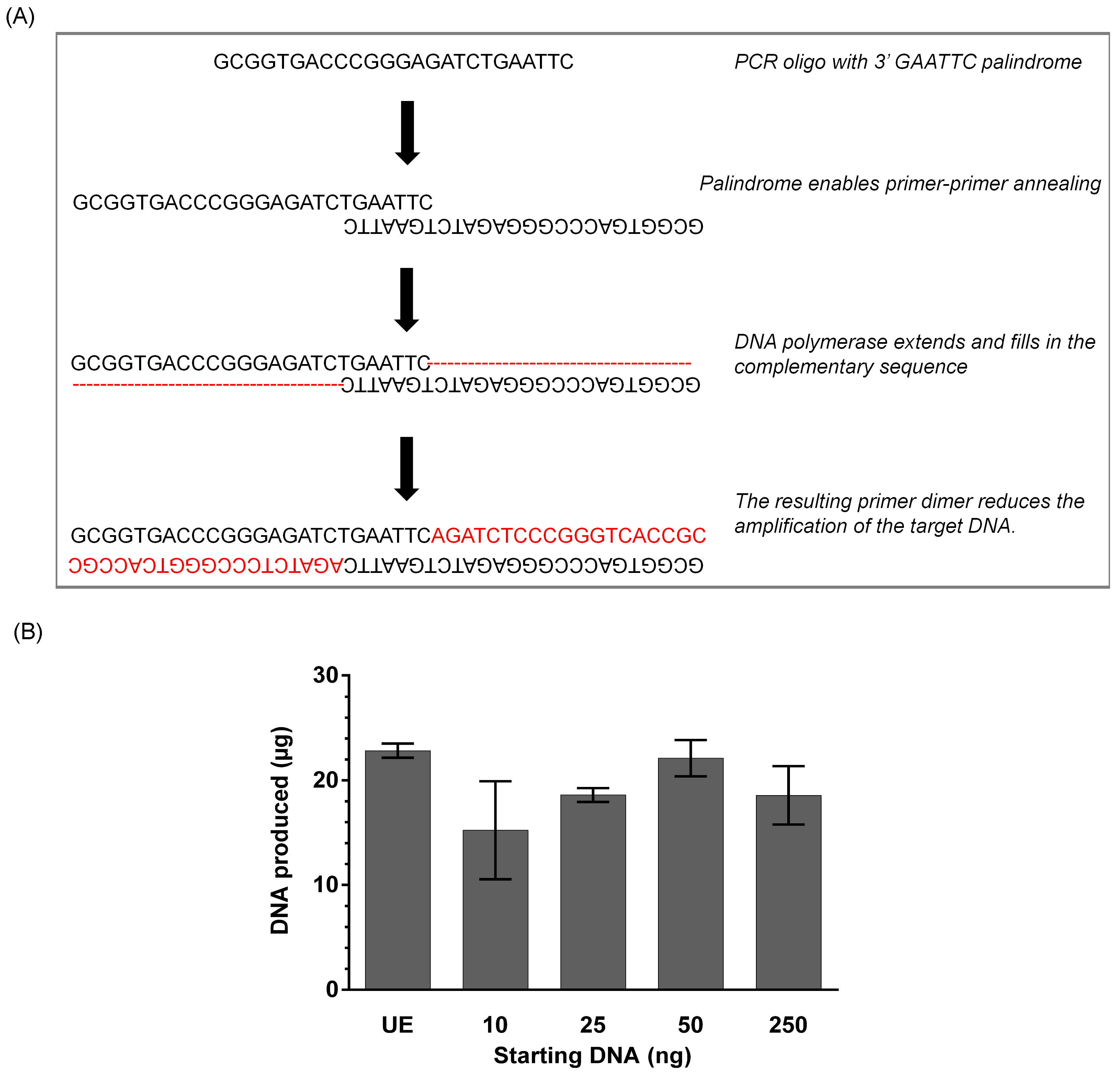

Supplement: Additional file 1: Figure S1 — The KLM-PCR protocol. (A) Modification of the universal adapter oligo sequence. The original LM-PCR oligo contained a palindrome at the 3′ end [17]. Prevention of dimerization through the disruption of the oligo palindrome increases the amount of available oligo for KLM-PCR amplification. dNTPs are now the limiting factor in the amplification. (B) NanoDrop quantification of the mean micrograms of DNA produced after KLM-PCR. Bars represent the mean of duplicate experiments from amplification of 10, 25, 50 and 250 ng amounts of pre-enriched starting DNA, or 10 ng of unenriched (UE) DNA. Error bars show standard deviation (n = 2). [file 1756-8935-6-17-S1.tiff]

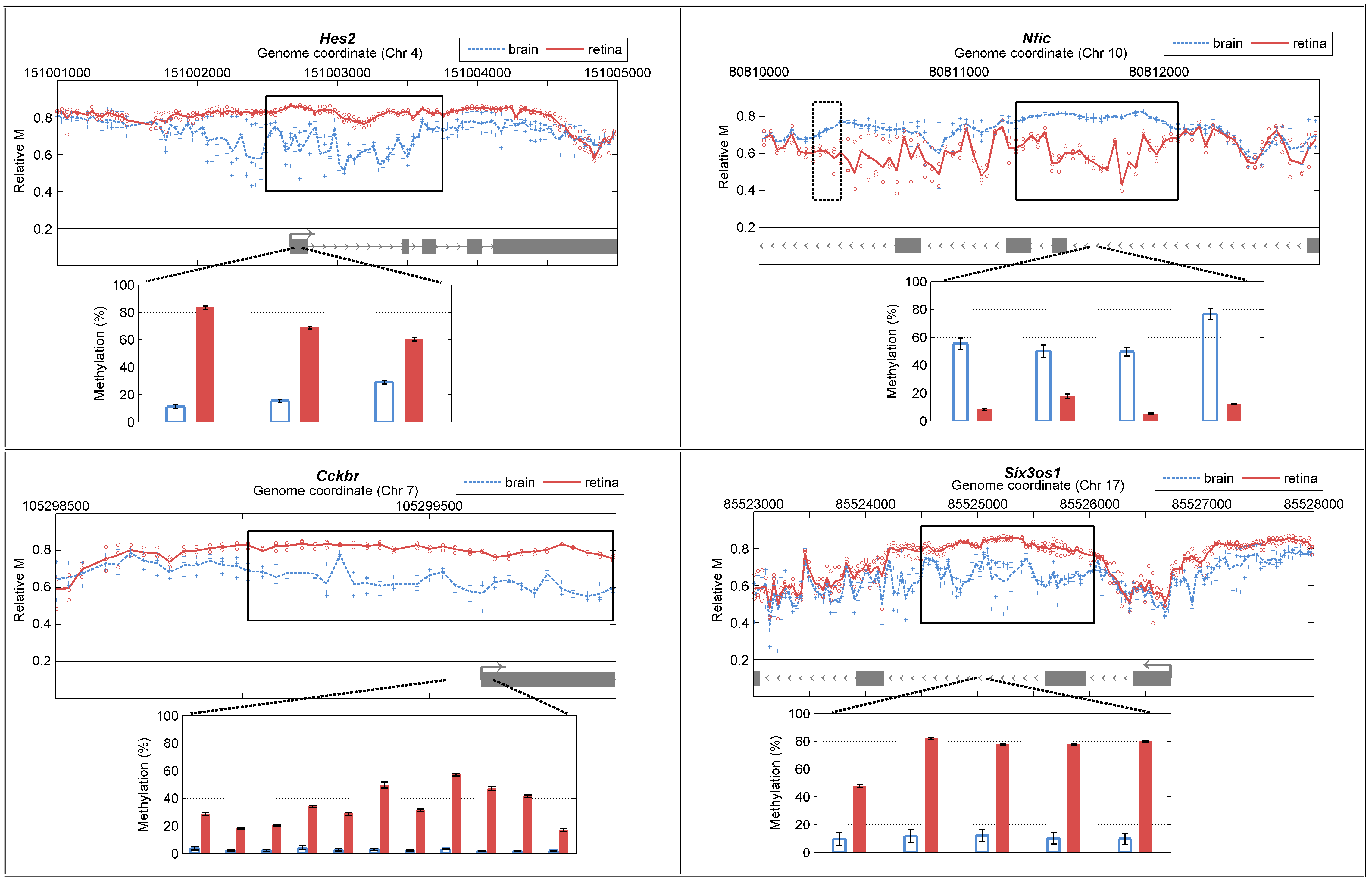

Supplement: Additional file 3: Figure S2 — Relative CpG methylation in the retina (red) and brain (blue) of the four other top T-DMRs (black boxes) evaluated using MeKL-chip (top plots) and pyrosequencing validation of the differential methylation (bottom graphs). See Figure 2C for description of MeKL-chip results. Pyrosequencing of CpGs within the T-DMR confirmed differential methylation (P < 0.001, Student’s two-tailed, paired t-test) between the retina (red bars) and brain (blue bars) in a second cohort of mice. Error bars show the 95% CI (n = 5). [file 1756-8935-6-17-S3.tiff]

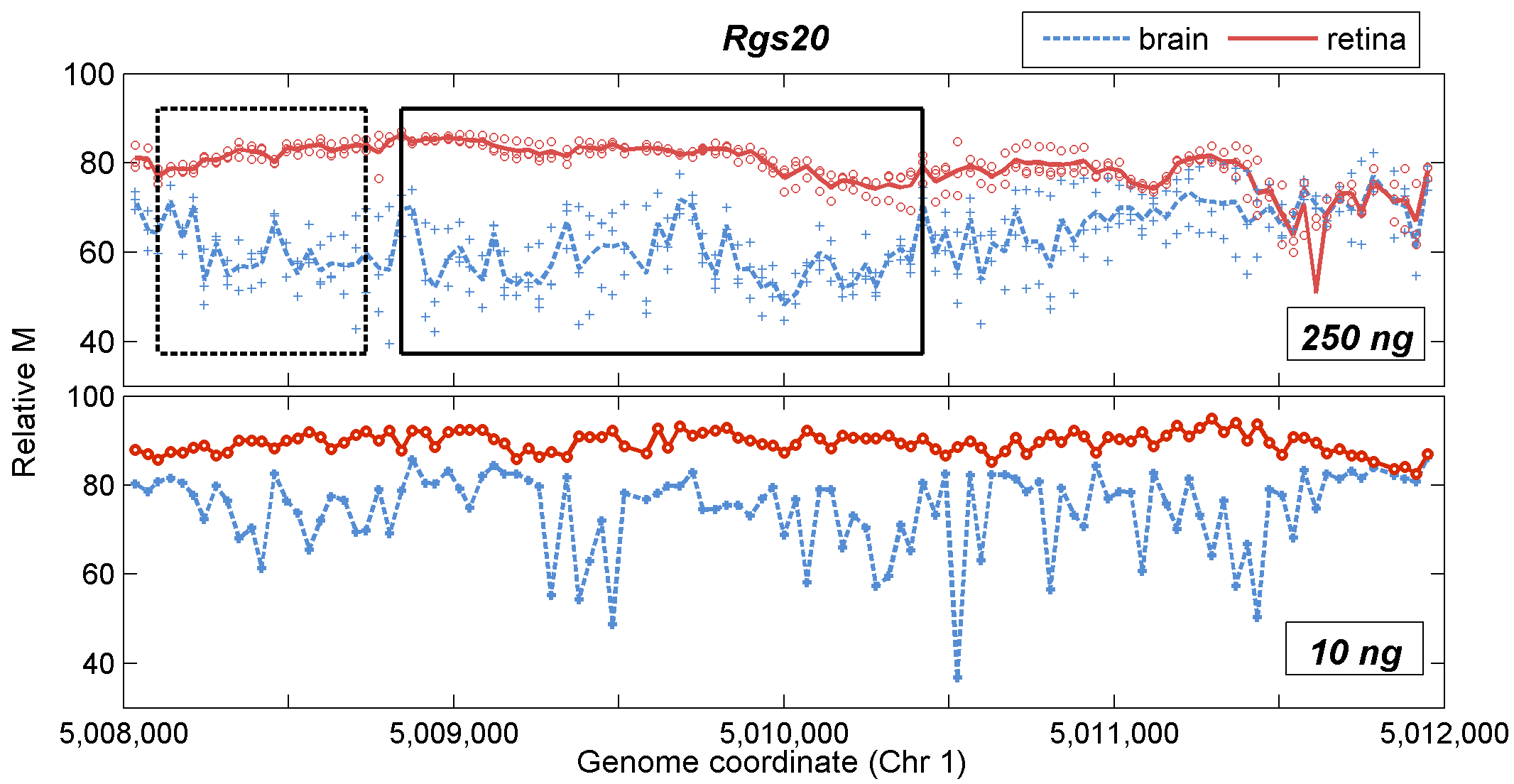

Supplement: Additional file 5: Figure S4 — MeKL-chip CpG site methylation profiles of the Rgs20 region identified as a T-DMR (highest ranked, P < 10-16, black box; lower ranked, P < 0.0091, dashed box) for the 250-ng high-input samples in the brain (blue) and retina (red) (top plot) as previously shown in Figure 2. The 10 ng low-input sample at the same region of Rgs20 is shown for direct comparison (lower plot) in brain (blue) and retina (red). Each point is the relative percentage methylation for 1 probe in 1 sample. The 250 ng plot contains biological triplicates and blue and red lines show the average methylation. The 10 ng plot contains one biological sample. The T-DMRs are still detectable in the 10 ng low-input sample. [file 1756-8935-6-17-S5.tiff]
