# Supplementary material for: A novel methyl-binding domain protein enrichment method for identifying genome-wide tissue-specific DNA methylation from nanogram DNA samples
Source: Epigenetics Chromatin. 2013 Jun 7;6:17. doi: 10.1186/1756-8935-6-17 (PMC3680319; doi:10.1186/1756-8935-6-17)
Supplement: Additional file 2: Table S1 — Top 20 differentially methylated regions identified between mouse retina and brain from 250 ng of starting DNA. Table S2. PCR amplification primers used for pyrosequencing the top five DMRs identified using MeKL-chip. Table S3. Sequencing primers used for pyrosequencing validation of the top five DMRs identified using MeKL-chip. [file 1756-8935-6-17-S2.docx]

**Additional File**

**Supplementary Table 1.** Top 20 differentially methylated regions identified between mouse retina and brain from 250 ng starting DNA.

| \| **Genome Browser Coordinates (mm8)** \| **Gene Symbol** \| **# Probes in DMR** \| **Distance to nearest CpG Island (kb)** \| **Hypermethylated Tissue** \| **% DNA Methylation Difference** \| \| --- \| --- \| --- \| --- \| --- \| --- \| \| chr1: 5008842-5010420 \| *Rgs20* \| 45 \| 0 \| Retina \| 23.2 \| \| chr4: 151002490-151003752 \| *Hes2* \| 37 \| 0 \| Retina \| 16 \| \| chr10: 80811285-80812095 \| *Nfic* \| 24 \| 11.3 \| Brain \| 19.1 \| \| chr7: 105299015-105299993 \| *Cckbr* \| 28 \| 0 \| Retina \| 18.4 \| \| chr17: 85524494-85526040 \| *Six3os1* \| 42 \| 0.25 \| Retina \| 14.8 \| \| chr3: 67551383-67552253 \| *Lxn/Gfm1* \| 26 \| 0 \| Retina \| 20.3 \| \| chr4: 147779558-147780611 \| *Casz1* \| 31 \| 1.8 \| Brain \| 10.3 \| \| chr2: 160056609-160057716 \| *Mafb* \| 31 \| 0 \| Retina \| 16 \| \| chr14: 35967758-35969018 \| *Lrit1* \| 37 \| 77.5 \| Brain \| 11.9 \| \| chr10: 75110765-75111777 \| *Cabin1* \| 29 \| 0 \| Retina \| 20.1 \| \| chr5: 105971779-105972934 \| *Lrrc8d* \| 34 \| 0 \| Retina \| 13.2 \| \| chr7: 18551543-18552977 \| *Ercc2/Klc3* \| 42 \| 0 \| Brain \| 8.5 \| \| chr7: 18548267-18549386 \| *Ercc2* \| 33 \| 3.2 \| Brain \| 7.4 \| \| chr11: 69781551-69782594 \| *Cldn7* \| 29 \| 0 \| Retina \| 19.3 \| \| chr4: 137602530-137603521 \| *Pink1* \| 28 \| 4.1 \| Brain \| 14.4 \| \| chr17: 25522323-25523682 \| *Metrn* \| 39 \| 0.2 \| Brain \| 12.8 \| \| chr16: 92387196-92388249 \| *Clic6* \| 31 \| 0 \| Retina \| 15.7 \| \| chr2: 148098604-148099585 \| *Thbd* \| 29 \| 0 \| Brain \| 17.8 \| \| chr2: 28791755-28792907 \| *1700101E01Rik* \| 34 \| 51.6 \| Brain \| 9.2 \| \| chr17: 71654353-71655565 \| *BC027072* \| 35 \| 31.8 \| Brain \| 10.4 \| |
| --- | --- | --- | --- | --- | --- | --- | --- | --- | --- | --- | --- | --- | --- | --- | --- | --- | --- | --- | --- | --- | --- | --- | --- | --- | --- | --- | --- | --- | --- | --- | --- | --- | --- | --- | --- | --- | --- | --- | --- | --- | --- | --- | --- | --- | --- | --- | --- | --- | --- | --- | --- | --- | --- | --- | --- | --- | --- | --- | --- | --- | --- | --- | --- | --- | --- | --- | --- | --- | --- | --- | --- | --- | --- | --- | --- | --- | --- | --- | --- | --- | --- | --- | --- | --- | --- | --- | --- | --- | --- | --- | --- | --- | --- | --- | --- | --- | --- | --- | --- | --- | --- | --- | --- | --- | --- | --- | --- | --- | --- | --- | --- | --- | --- | --- | --- | --- | --- | --- | --- | --- | --- | --- | --- | --- | --- | --- |

**Supplementary Table 2.** The PCR amplification primers used for pyrosequencing of the top 5 DMRs identified using MeKL-chip.

| Gene | Forward Primer | Reverse Primer (5´ Biotinylated) | Annealing Temp. (°C) |
| --- | --- | --- | --- |
| *Rgs20* | TGGGGTTTTGTGAATGAAGAGAT | ATACACTCCACCCTACCAT | 61 |
| *Hes2* | TTGGGGTTTAGAGGAGTAGT | TAAACCCCTCACATTAAATACTCCATA | 60 |
| *Nfic* | GTGTTTGGAAAGAGTATAGAGTTAGAG | ACTACTTCCAACCCTATAACACT | 61 |
| *Cckbr* | GGTATGAGAGGTGGGTAGAAAA | CCACCAACCTTCCCTTAAAC | 60 |
| *Six3os1* | GGATGTGGGGGGTGGAAG | CCCTACTAAAACCCCACCATT | 60 |

**Supplementary Table 3.** The sequencing primers used for pyrosequencing validation of the top 5 DMRs identified using MeKL-chip.

| Gene | Sequencing Primers |
| --- | --- |
| *Rgs20* | GAGAGGAGTTGGTGT |
|  | GTTTTTTTATTTTAAATAGTGGTTT |
| *Hes2* | AAGTTGAAAATGAGATGAAT |
| *Nfic* | GAGTTTTGATGGTTATATGATA |
| *Cckbr* | AGATATTTTTAAGAGAAGGAGA |
|  | TTTGTTTGGAGTGTAGT |
|  | TTTGGAGTTAAGGAGG |
|  | AGTTATTTGGTGGGATAAA |
| *Six3os1* | GGGGTTTATTAGGTAGA |

**Supplementary Figure Legends**

**Supplementary Figure 1.** The KLM-PCR protocol. (A) Modification of the universal adapter oligo sequence. The original LM-PCR oligo contained a palindrome at the 3ʹ end [[9](#_ENREF_9)]. Prevention of dimerization through the disruption of the oligo palindrome increases the amount of available oligo for KLM-PCR amplification. dNTPs are now the limiting factor in the amplification. (B) NanoDrop quantification of the mean µg DNA produced after KLM-PCR. Bars represent the mean of duplicate experiments from amplification of 10, 25, 50 and 250 ng pre-enriched starting DNA, or 10 ng of unenriched (UE) DNA. Error bars show standard deviation (n=2).

**Supplementary Figure 2.** CpG methylation of the 4 other top T-DMRs (black boxes) between retina (red) and brain (blue) using MeKL-chip (top plots) and pyrosequencing validation of the differential methylation (bottom graphs). See Figure 2C for description of MeKL-chip results. Pyrosequencing of CpGs within the T-DMR confirmed differential methylation (*p* < 0.001, Student’s two-tailed, paired t-test) between the retina (red bars) and brain (blue bars) in a second cohort of mice. Error bars, 95% CI (n=5).

**Supplementary Figure 3.** Pre-hybridization validation of enrichment from low-input (10, 25 and 50 ng) enriched DNA from retina and brain samples. The mixed effects regression model with a random intercept for the measures from triplicate QPCR of 2 PCR amplifications of the same sample were used to calculate the mean difference and standard error in fold enrichment. (a) Post-enrichment, *Rbp3* (grey bars) and *Rho* (black bars) were enriched for methylated DNA in the brain samples for all amounts of starting DNA, whereas no enrichment was observed in unenriched (UE) DNA. (b) Post-KLM-PCR, QPCR showed maintenance of the enrichment pattern for methylation in the brain samples for *Rho* (black bars) and *Rbp3* (grey bars) and no enrichment of the UE samples.

**Supplementary Figure 4.** MeKL-chip CpG site methylation profiling of the *Rgs20* region identified as a T-DMR (highest ranked, *p* < 1E-16, black box; lower ranked, *p* < 0.0091, dashed box) for the 250 ng high-input samples in brain (blue) and retina (red) (top plot) as previously shown in Figure 2. The 10 ng low-input sample at the same region of *Rgs20* is shown for direct comparison (lower plot) in brain (blue) and retina (red). Each point is the relative percentage methylation for 1 probe in 1 sample. The 250 ng plot contains biological triplicates and blue and red lines show the average methylation. The 10 ng plot contains one biological sample. The T-DMRs are still detectable in the 10 ng low-input sample.
